# Characteristics of mesoscale eddies in the Mozambique Channel

**DOI:** 10.1371/journal.pone.0302367

**Published:** 2024-04-29

**Authors:** Linfei Bai, Guohao Zhu, Haojie Huang, Liqiong Zhang, Haibin LÜ, Yinyi Zhang

**Affiliations:** 1 Jiangsu Key Laboratory of Marine Bioresources and Environment /Jiangsu Key Laboratory of Marine Bio-technology, Jiangsu Ocean University, Lianyungang, Jiangsu Province, China; 2 School of Marine Technology and Geomatics, Jiangsu Ocean University, Lianyungang, Jiangsu Province, China; 3 Lianyungang Meteorological Bureau, Lianyungang, Lianyungang, Jiangsu Province, China; 4 Co-Innovation Center of Jiangsu Marine Bio-industry Technology, Jiangsu Ocean University, Lianyungang, Jiangsu Province, China; Universidade de Aveiro, PORTUGAL

## Abstract

The mesoscale eddy characteristics of the Mozambique Warm Current were investigated by detecting and tracking satellite altimetry data from 2010 to 2019. A total of 1,086 eddies were identified in the Mozambique Channel, comprising 509 cyclonic eddies and 577 anticyclonic eddies. The results revealed that the bay area on the northwest coast of Madagascar was the main hotspot of eddy generation, and the mean amplitude and radius of the anticyclonic eddies in the Mozambique Channel were 24.23 cm and 82.7 km, respectively, which are larger than those of the cyclonic eddies. Local wind forcing had a significant impact on the formation of mesoscale eddies in the Mozambique Channel. In winter, the wind stress in the northern and southern areas of the Mozambique Channel exhibited a strong correlation with the distribution of eddy kinetic energy (EKE), where both monsoonal winds in the north and trade winds in the south could facilitate mesoscale anticyclonic eddy formation. In addition, the variability in the number of anticyclonic and cyclonic eddies in the Mozambique Channel may have exerted a significant influence on the seasonal anomalous fluctuations in local sea surface temperatures (SSTs). This study presented a novel analysis of the mesoscale eddy characteristics in the Mozambique Channel.

## Introduction

The Mozambique Channel is a slender waterway that emerges between Madagascar Island and the African continent, flanked on its northern side by the Comoros Islands and on its southern side by the Agulhas Current [1]. The Southern Equatorial Current in northern Madagascar splits into the North East African Coastal Current and the Mozambique Warm Current near the 11° S latitude, flowing south into the Mozambique Channel [2]. The Mozambique Warm Current is a western boundary current that is unlike any other [3]. It is an ocean circulation dominated by large anticyclonic eddies that travel southward through the channel, where one of the strongest eddy activity regions in the world occurs [4–6].

The formation of mesoscale anticyclonic eddies to the west of the tip of Madagascar is linked to barotropic instability related to South Equatorial Current, while mesoscale cyclonic eddies tend to occur near the Comoros Islands due to strong baroclinic instability, resulting in dipole eddies in the waters northwest of Madagascar [7–9]. Influenced by the seasons, the maximum (minimum) eddy occurs in the southern channel from winter to spring (summer to autumn) [9].

This region exhibits both higher eddy kinetic energy and a higher sea level height [10]. In addition, mesoscale characteristics have a great influence on the heat storage rate of the mixed layer and affect the flux exchange between the local ocean and the atmosphere [11–13]. The interaction between the ocean and atmosphere in the Mozambique Channel exerts a significant influence on tropical weather systems in the region, with severe tropical cyclones and mesoscale convective systems frequently occurring due to abnormally warm sea temperatures and other conducive conditions for cyclone formation [14,15]. Previous studies have demonstrated the crucial role of mesoscale anticyclonic eddies in tropical cyclone development, leading to significant enhancement of tropical cyclone research [11,16,17]. For example, Tropical Storm Deliwe in 2014 rapidly intensified through these upper ocean eddies with a high heat content and enthalpy flux and formed a unique reverse track [18]. In addition, eddies can generate near-inertial internal waves, which in turn exert force and torque on the tendon legs of offshore platforms [19], thus affecting the construction and subsequent maintenance of marine facilities [20,21].

Wind stress and its curl are fundamental driving forces for oceanic dynamic processes, with local wind stress and its curl playing a crucial role in sea-air interactions and momentum exchange [8]. The weakening of the Southern Equatorial Current and Mozambique Channel flow due to trade winds could result in a reduction in the Agulhas Spill in the southern Mozambique Channel [22]. Moreover, the local wind fields and the Mozambique Channel eddies are likely to exert significant control over the east–west gradient of sea surface temperature (SST) in the Mozambique Channel [23].

Swart et al [24] provided a (about two weeks) hydrographic station data study and analysis of an observed large warm eddy with a diameter of more than 200 km, focusing on the abnormal changes in its heat and salinity and the impact on the water mass of the Agulhas Current. However, there are few studies on the characteristics and effects of mesoscale eddies in the Mozambique Channel at the seasonal scale, so we conducted the following studies based on ten years of observational data using the latest eddy detection and tracking algorithms.

We investigated the generation and trajectory of eddies in the Mozambique Channel using state-of-the-art satellite altimeter observations and eddy tracking algorithms [25]. Initially, we analyzed 10 years (2010–2019) of sea level data to obtain basic statistics. The objectives of this study were to (1) examine the spatiotemporal variation in mesoscale eddies in the Mozambique Channel; (2) comprehend the impact of monsoons on mesoscale eddies in this region; and (3) investigate the effect of mesoscale eddies on local SSTs. This manuscript is structured as follows: In Data and Methods section, the data and methodology is outlined; In Results section, our main statistical findings are presented; Relevant discoveries are discussed in Discussion section; and finally, we summarize the conclusions.

## Data and methods

### Study area

**Fig 1:**
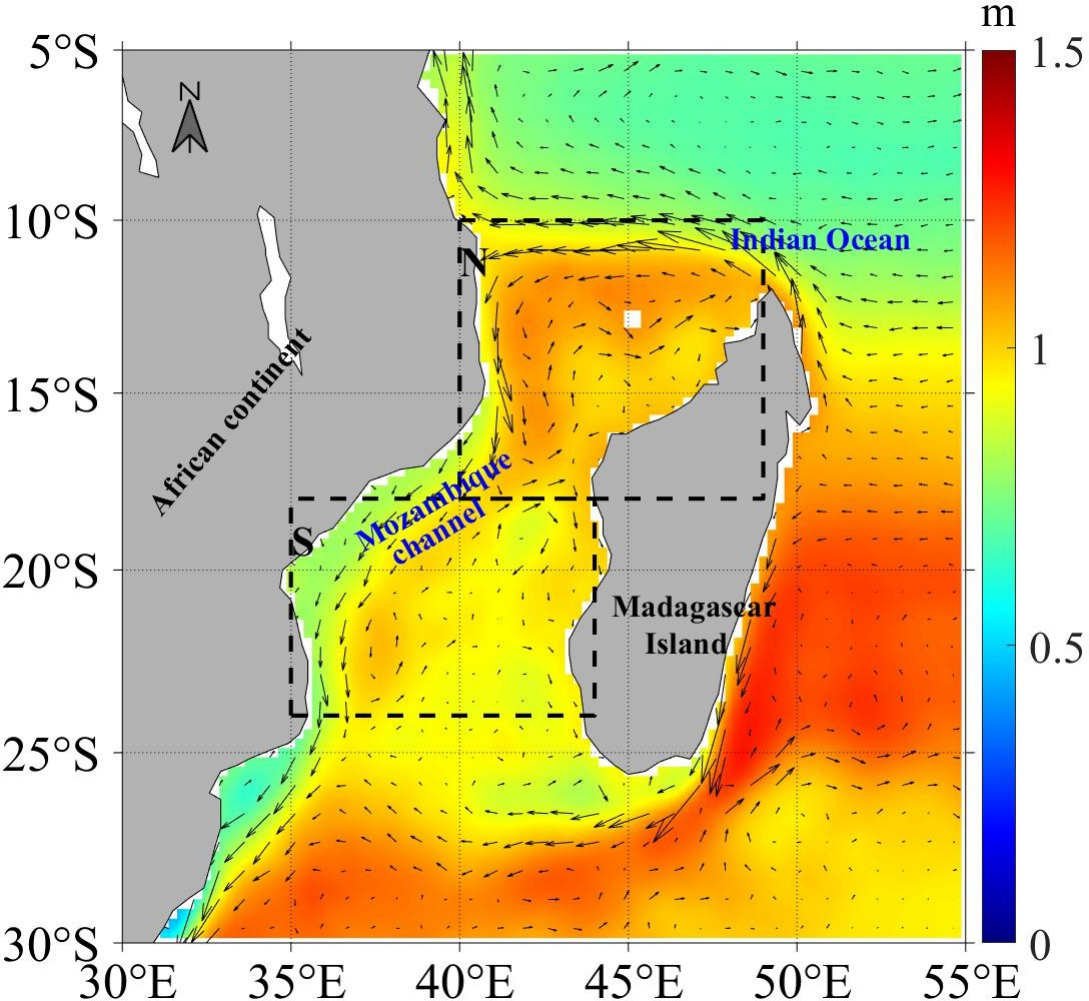
Composite of the sea surface height and current surrounding the Mozambique Channel from 2010 to 2019. The study areas are indicated by dashed black squares, i.e., Box N: 10°S–18°S, 40°E–49°E; Box S: 18°S–24°S, 35°E–44°E.

The study area is situated in the Mozambique Channel, nestled in the southeastern region of the African continent (Fig 1). Box N (10°S-18°S, 40°E-49°E) and Box S (18°S-24°S, 35°E-44°E) are defined to differentiate the distinct monsoon regions in the northern and southern parts of the Mozambique Channel, which are indicated by dashed black squares in Fig 1.

### Data

Eddy detection and tracking data, the flow field of the composite and the "all-satellite" day delay time (DT) data from the 2018 version of the Data Unification and Altimeter Combination System (DUACS) (https://cds.climate.copernicus.eu/) are obtained. The selected time span is from January 1, 2010, to December 31, 2019.

The ERA5 datasets from the European Centre for Medium-Range Weather Forecasts (ECMWF, https://cds.climate.copernicus.eu/) provide the atmospheric reanalysis data, calculating the composite wind component over ten years.

The remote sensing system (RSS, http://www.remss.com/measurements/). provides the daily SST data.

### Methods

#### Eddy detection algorithm

The eddy current testing method is based on the geometric velocity vector, and the eddy current center point is determined by satisfying four constraints. The eddy size is determined by the closed contour of the flow function field, while the trajectory of the eddy is retrieved through a comparison of its continuous time steps [25,26]. This is consistent with the definition of the basic characteristics of eddy currents in previous studies [27]. In this algorithm, the contour line of the flow function is used to define the boundary of the eddy, which is the outermost enclosing contour line around the center point. Finally, considering the splitting and merging of the eddies, the trajectories of the vortices are calculated.

The amplitude of the eddy is determined by calculating the height difference between the sea surface height (SSH) inside the eddy and the SSH at the edge.

$$\text{The}\;\text{amplitude},\text{A}=\left|{\text{SSH}}_{\text{ce}}-{\text{SSH}}_{\text{ed}}\right|$$ (1)

where SSHce and SSHed are the SSH at the center and at the edge of the eddy, respectively.

The radius of the eddy, denoted as R, is defined as the average distance between each edge point of the eddy and its center.

#### Eddy kinetic energy (EKE)

Eddy kinetic energy (EKE) is an important physical quantity that determines the activity of eddies and is an important variable for exploring the instability of oceans by using the energetics principle [28].

$$EKE=0.5\left(u\prime^{2}+v\prime^{2}\right)$$ (2)

where the latitudinal velocity component is denoted as u, while the longitudinal velocity component is represented by v.

## Results

### Path and distribution of ocean eddies

**Fig 2:**
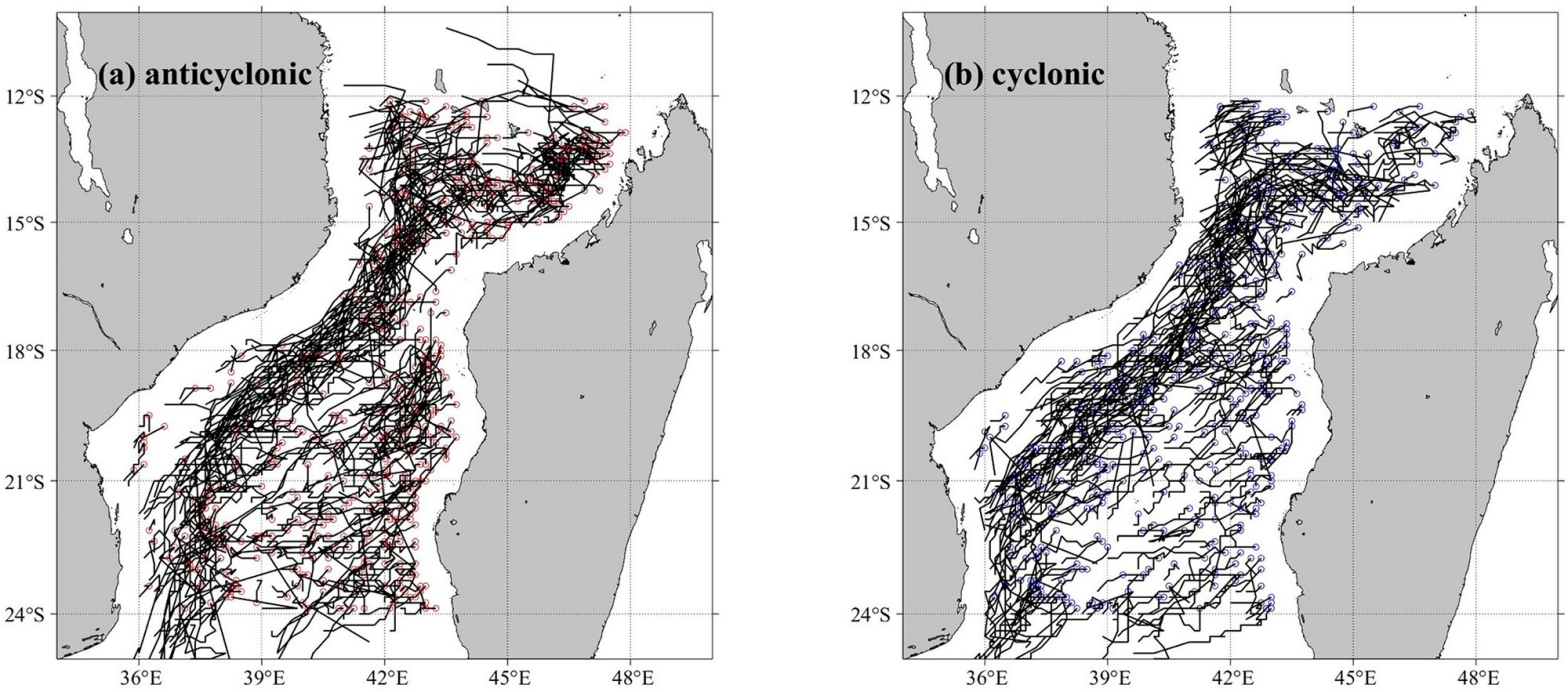
Origin and developmental trajectories of anticyclonic (a) and cyclonic (b) eddies in the Mozambique Channel.

The path and distribution analysis of ocean eddies based on satellite altimeter data are presented in Fig 2 by the automatic eddy detection and tracking algorithm. Mesoscale eddies are prevalent in the Mozambique Channel. From 2010 to 2019, the tracking algorithm identified 1,086 eddies in the Mozambique Channel, comprising 509 cyclonic eddies and 577 anticyclonic eddies. Fig 2a shows the origin and developmental trajectories of the anticyclonic eddies, which originate primarily off the northern and western coasts of Madagascar and move southward along the western boundary of the channel. According to previous research [29,30], anticyclonic eddies are the dominant eddies in the Mozambique Channel, and cyclonic eddies are generated through interactions with them. Fig 2b shows the origins and developmental trajectories of cyclonic eddies, showing that both types of eddies follow similar tracks and distributions in the Mozambique Channel.

**Fig 3:**
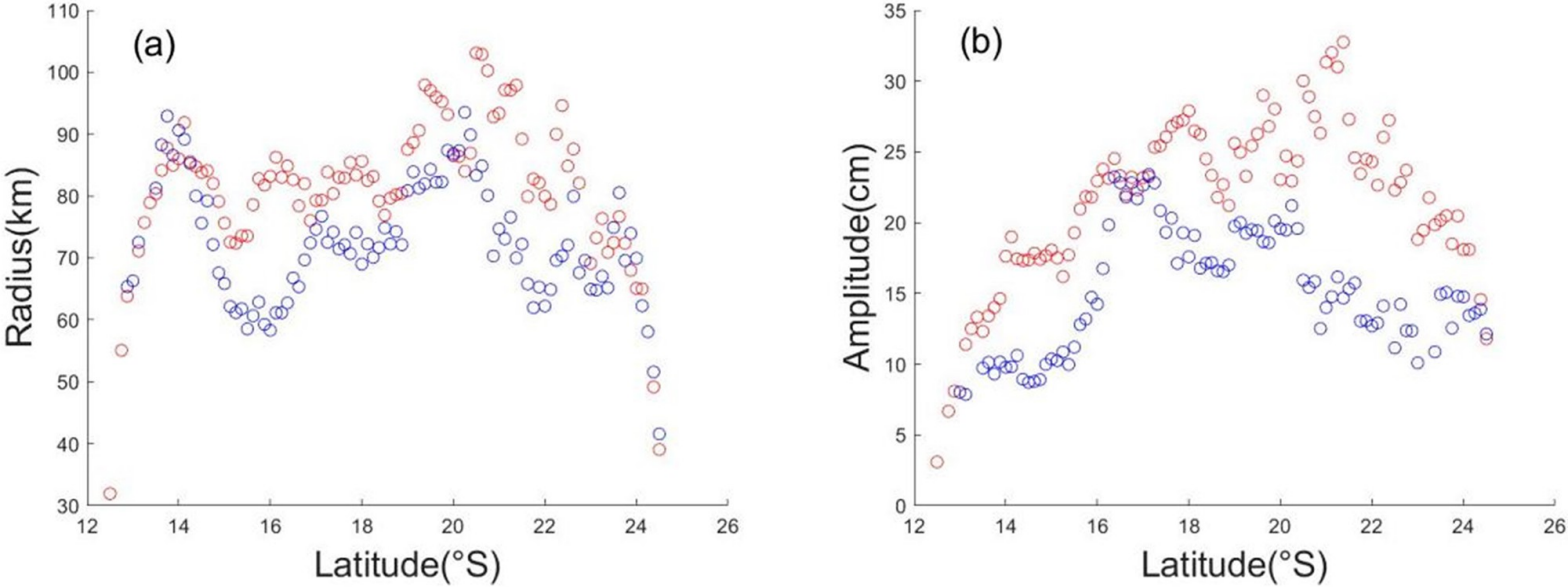
The (a) radius (unit: km) and (b) amplitude (unit: cm) of the eddies corresponding to latitudinal variations. The red (blue) circles are anticyclonic (cyclonic) eddies.

The latitudinal variation in the eddy characteristics in the Mozambique Channel is shown in Fig 3. Cyclonic and anticyclonic eddies have average amplitudes of 24.23 cm and 15.51 cm, respectively, with corresponding average radii of 82.7 km and 69.27 km, respectively, in the Mozambique Channel. These findings confirm that anticyclonic eddies demonstrate a higher level of dynamic dominance in comparison to cyclonic eddies in the Mozambique Channel.

The characteristic radii and amplitudes of the eddies in the Mozambique Channel have different trends with latitude, and the characteristic radius is obviously more restricted by the channel topography (Fig 3a). When the anticyclonic and cyclonic eddies pass through the narrow northern part of the Mozambique Channel between 10°S and 18°S, their radii first decrease and then increase with increasing latitude (Fig 3a), while their amplitudes continue to increase (Fig 3b). However, south of 22°S, the eddies began to dissipate, and the overall amplitude decreases (Fig 3b). The eddies basically accumulate energy under the influence of the equatorial current in the Mozambique Channel until they reach 22°S. The characteristic radii and amplitudes of the mesoscale eddies in the Mozambique Channel are consistent with previous observations of global eddies [4].

**Fig 4:**
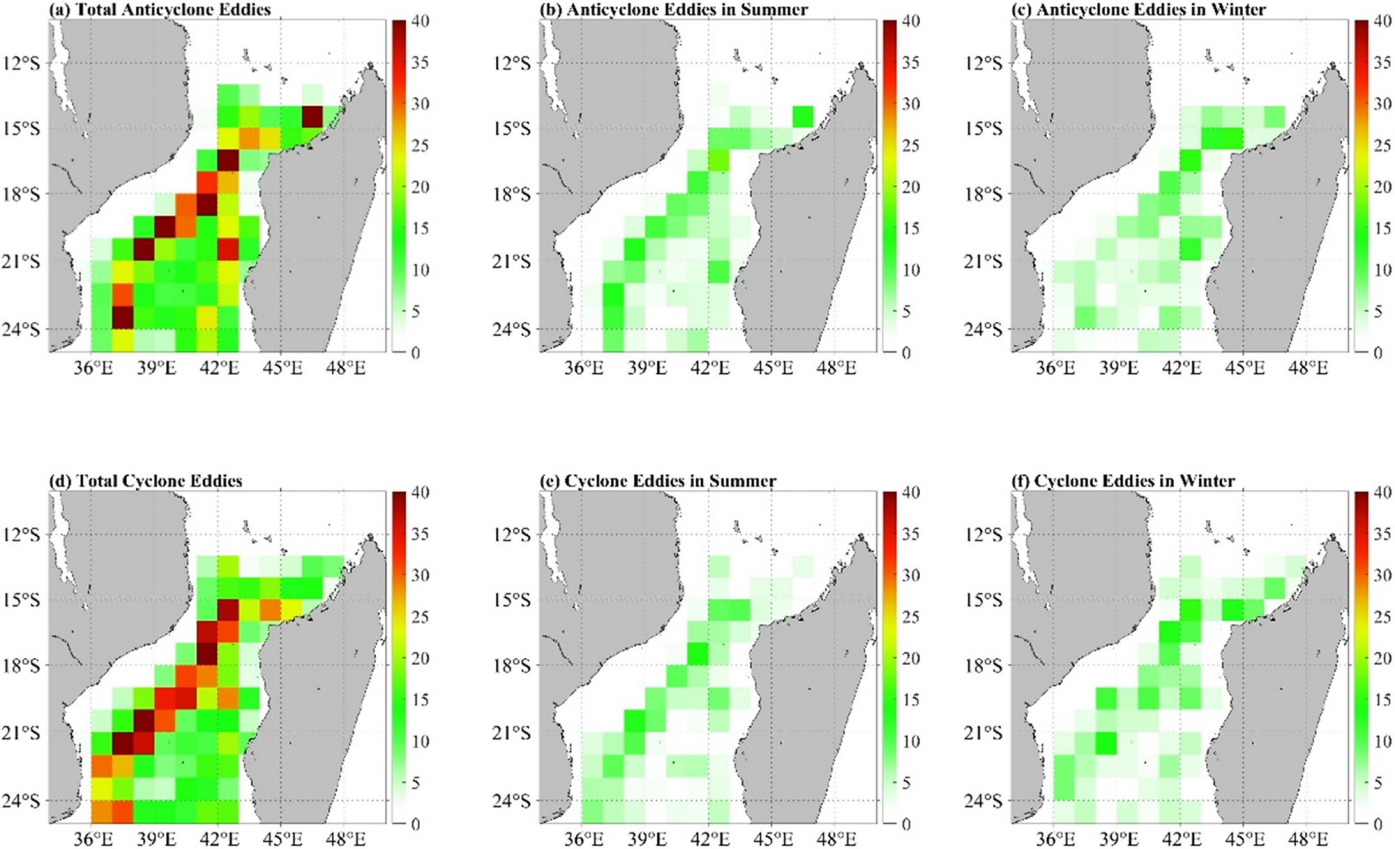
Spatial frequency distribution of anticyclonic and cyclonic eddies annually (a and d), in summer (b and e) and in winter (c and f).

The spatial frequency distributions of anticyclonic and cyclonic eddies as determined using the eddy detection algorithm is shown in Fig 4. The study area (34°E–50°E, 10°S-25°S) was divided into 1°× 1° grid boxes to record the geographical distribution of the ocean eddies, and the eddies falling in each grid box were counted to study the spatial distribution of the eddies. Here, the spatial frequency distributions of anticyclonic and cyclonic eddies at seasonal scales, namely, summer (December to February) and winter (May to July), were investigated. The mesoscale eddies were mainly concentrated in the offshore areas of the Mozambique Channel, where the number of eddies in the western part of the Mozambique Channel was significantly higher than that in the eastern part (Fig 4a and 4d). In addition, the concentrated area of anticyclonic eddies in winter extended further north compared to that in summer, with approximately 65% of the winter anticyclonic eddies located north of 21°S (Fig 4c), while in summer, a dense eddy distribution persisted until the latitude of 25°S(Fig 4b). However, the seasonal variations did not exert any significant impact on the abundance of cyclonic eddies (Fig 4e and 4f).

### Seasonal and annual characteristics of the ocean eddies

During the study period, 509 cyclonic eddies and 577 anticyclonic eddies were detected in the Mozambique Channel, corresponding to eddy locations at 1-day intervals over the decade. For each location, similar to the previous method [33], the SST within the radius determined in the eddy detection algorithm is used for synthesis. Specifically, the ten-year daily average SST over the radius of the corresponding position of the mesoscale eddy is used. The δSST is obtained at the pixel level by averaging the location of the mesoscale vortices on the corresponding date. By calculating the difference between the decadal mean SST and δSST of the mesoscale eddy in the Mozambique Channel, and by calculating the age average and seasonal average, ΔSST of each location is obtained [25,31].

**Fig 5:**
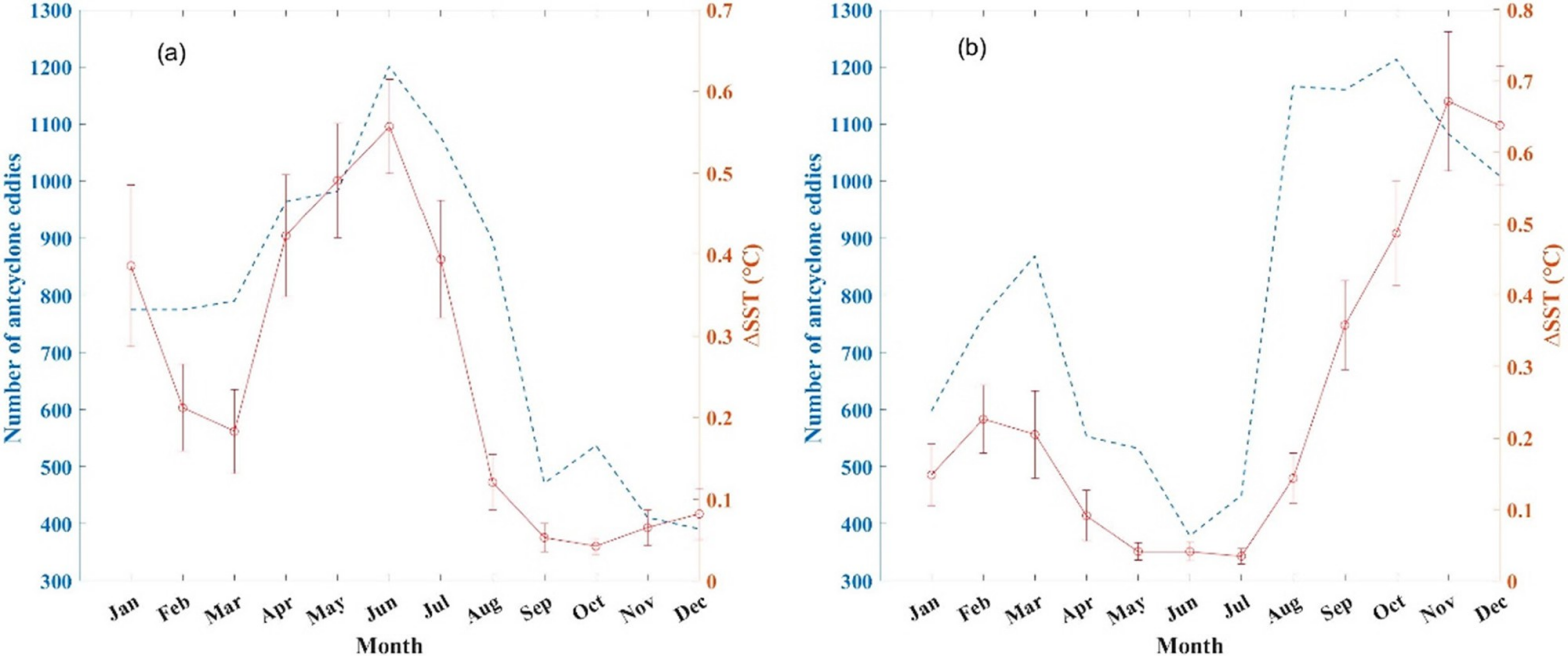
The seasonal statistics of anticyclonic (a) and cyclonic (b) eddies and ΔSST.

Fig 5 shows the relationship between the number of anticyclonic and cyclonic eddies and SST anomalies (the ΔSST of the cyclonic eddy is treated as an absolute value). The ΔSST variation is consistent with the number of eddies. During spring and summer (September to March) in the Mozambique Channel, there is a relatively low occurrence of anticyclonic eddies, resulting in a small temperature difference. The strait exhibits a relatively high frequency of cyclonic eddies, and a significant ΔSST contrast exists when the cold water in the deep layer is uplifted to the surface through the upwelling caused by cyclonic eddies (Fig 5b). However, the opposite is true in autumn and winter (April to August) for the anticyclonic eddies, and the ΔSST reaches 0.64°C (Fig 5a). The mesoscale features in the Mozambique Channel play a significant role in determining ocean heat storage rates. A previous study conducted on the southwest coast of Madagascar revealed that coastal anticyclonic eddies contributed to the intensification of marine heat waves in this region [32].

**Fig 6:**
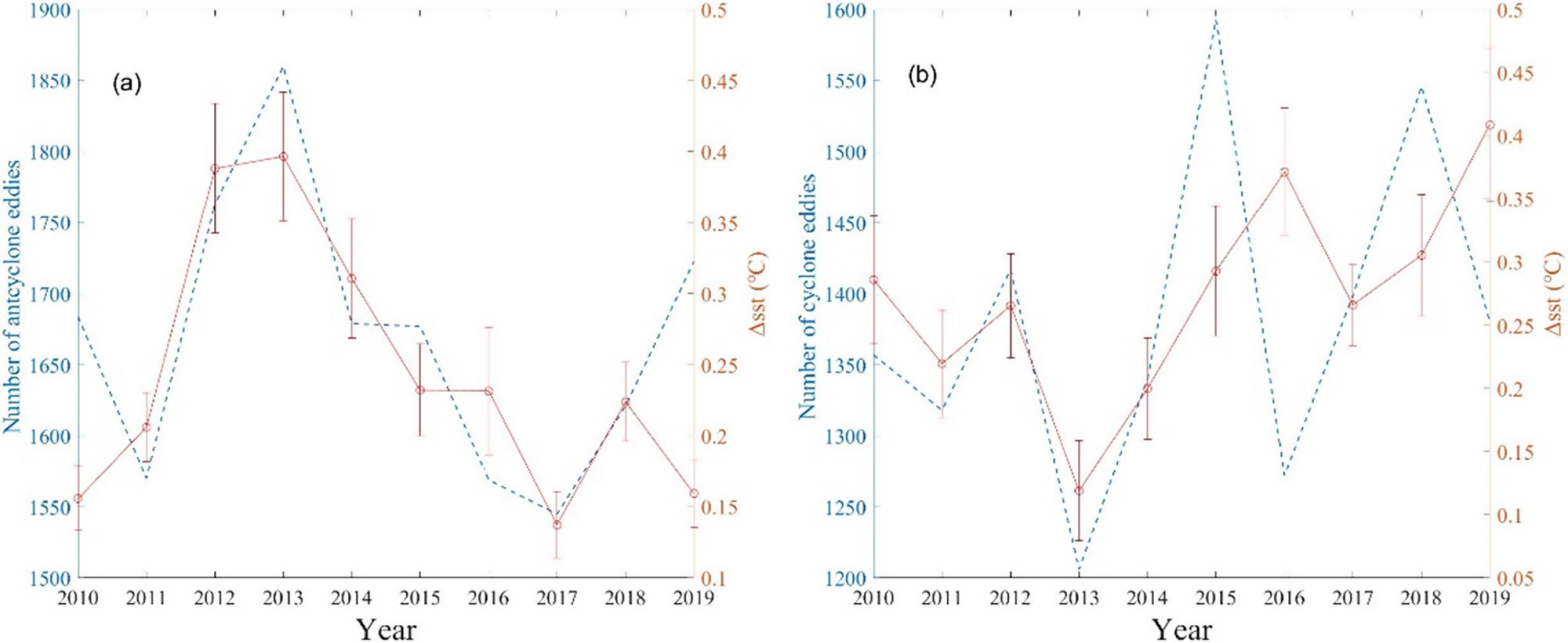
The annual statistics of anticyclonic (a) and cyclonic (b) eddies and ΔSST.

Furthermore, a robust correlation is observed between the number of anticyclonic eddies and ΔSST (Fig 6a). As the detected count of anticyclonic eddies increase to 1,774, the corresponding ΔSST outlier also exhibits an increase to 0.39 °C, reaching its peak in 2013. A decline is witnessed in subsequent years, with only 1,503 anticyclonic eddies being detected at the lowest point in 2017, accompanied by an outlier of ΔSST measuring 0.15 °C. However, no such significant correlation has been found in studies of interannual variations in cyclonic eddies (Fig 6b). This phenomenon may be due to the greater strength of the anticyclonic eddy than that of the cyclonic eddy in the Mozambique Channel (Fig 3), which exerts a major influence on the marine dynamics in the region [33].

## Discussion

### Role of local wind forcing

**Fig 7:**
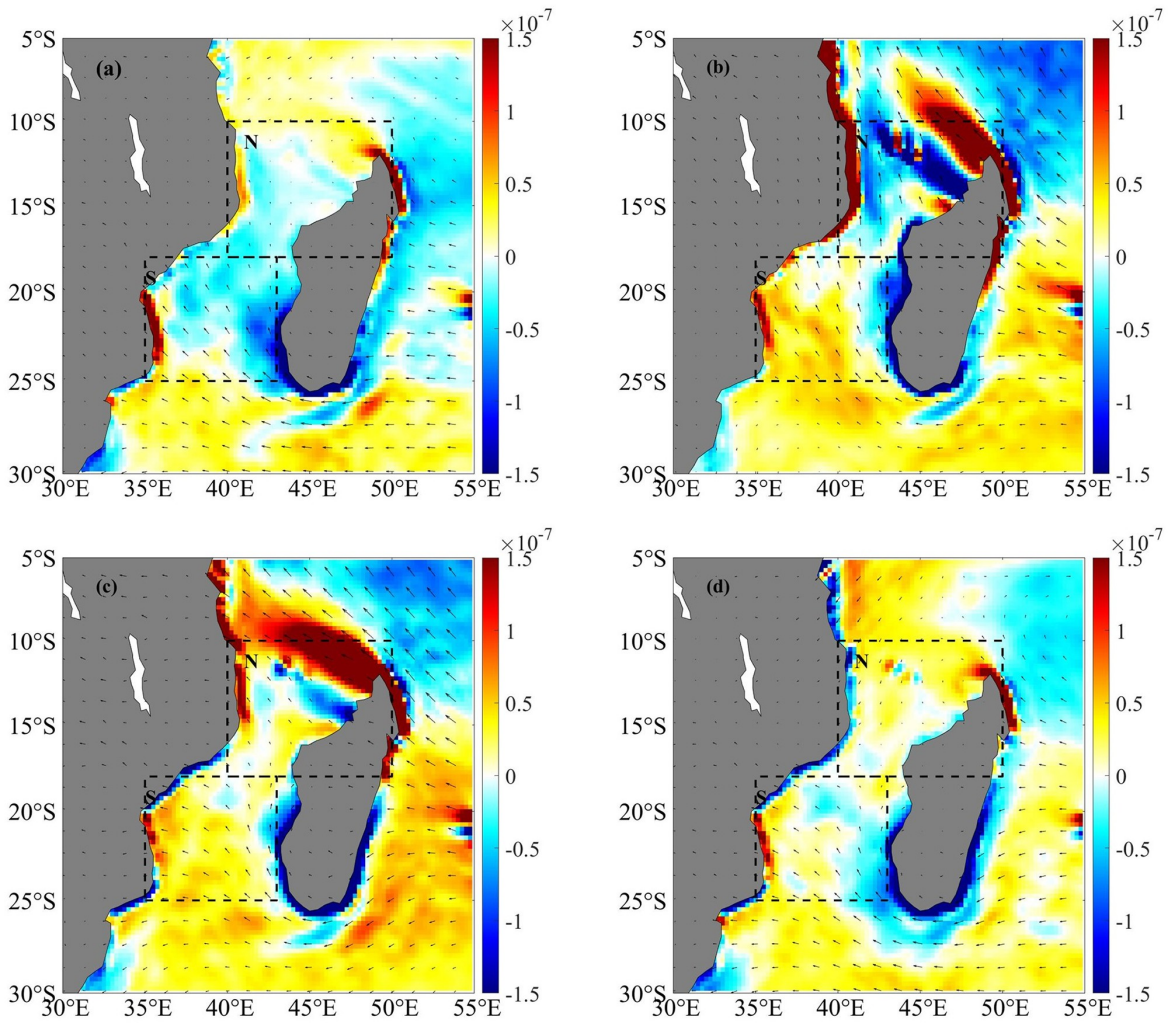
The average wind stress curl (N/m^3^) in the Mozambique Channel estimated from ERA5 reanalysis data for March (a), June (b), September (c), and December (d) from 2010 to 2019.

Since wind-induced instabilities could play a key role in the formation and development of ocean eddies, we investigate the influence of monsoons on Mozambique Channel eddies. The wind stress curl of the 10 m wind vector obtained from the ERA5 reanalysis data in March, June, September, and December is presented for analysis (Fig 7). The Mozambique Channel north of 18°S is primarily influenced by the winter northeast monsoon and summer southeast monsoon, while the region south of 18°S is dominated by year round southeast trade winds [34].

**Fig 8:**
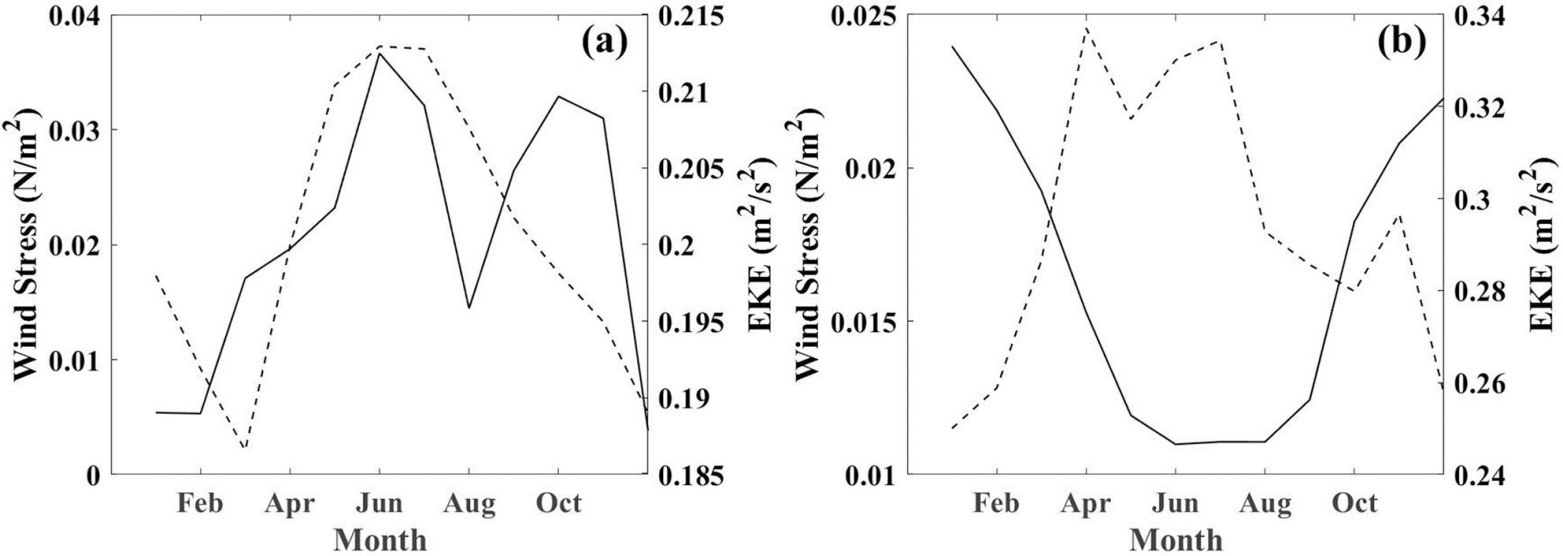
The monthly average climate changes in wind stress (N/m^2^) and EKE (m^2^/s^2^) are represented by dashed lines and solid lines, respectively (a is for Box N and b is for Box S, which are shown in Fig 1).

As anticipated, the wind stress curl exhibits a greater magnitude in autumn and winter than in spring and summer (Fig 8). The relation between the monthly EKE and wind stress in the northern and southern Mozambique Channel is depicted in Fig 8. In the northern Mozambique Channel, the wind stress is higher in winter (April to August). The correlation coefficient between winter wind stress and EKE in the northern Mozambique Channel is 0.983 (Fig 8a), which is very significantly over the 95% confidence interval, indicating that the combined enhancement of the northeast current of Madagascar and the local strong winds is suggested to be the primary factor facilitating the winter increase of anticyclonic eddies [1]. However, in the southern Mozambique Channel, the correlation coefficient between wind stress and EKE is -0.776 (Fig 8b). The weaker EKE may be due to stronger southeast trade winds and weaker EKE, which may slow the southward movement of the eddies (Fig 8b). In addition, strong south-western monsoons in northern Madagascar in winter produce strong positive wind stress curl in the region, which may cause instability in ocean currents flowing off the northern tip of Madagascar, resulting in a possible increase in the frequency of seasonal circulation, mainly manifested in a statistical increase in the number of anticyclones eddies[35]. This may be the reason that there are more anticyclonic eddies in winter and that the overall ΔSST is abnormally high (Fig 5a).

### Effect of ocean eddies on local SST

**Fig 9:**
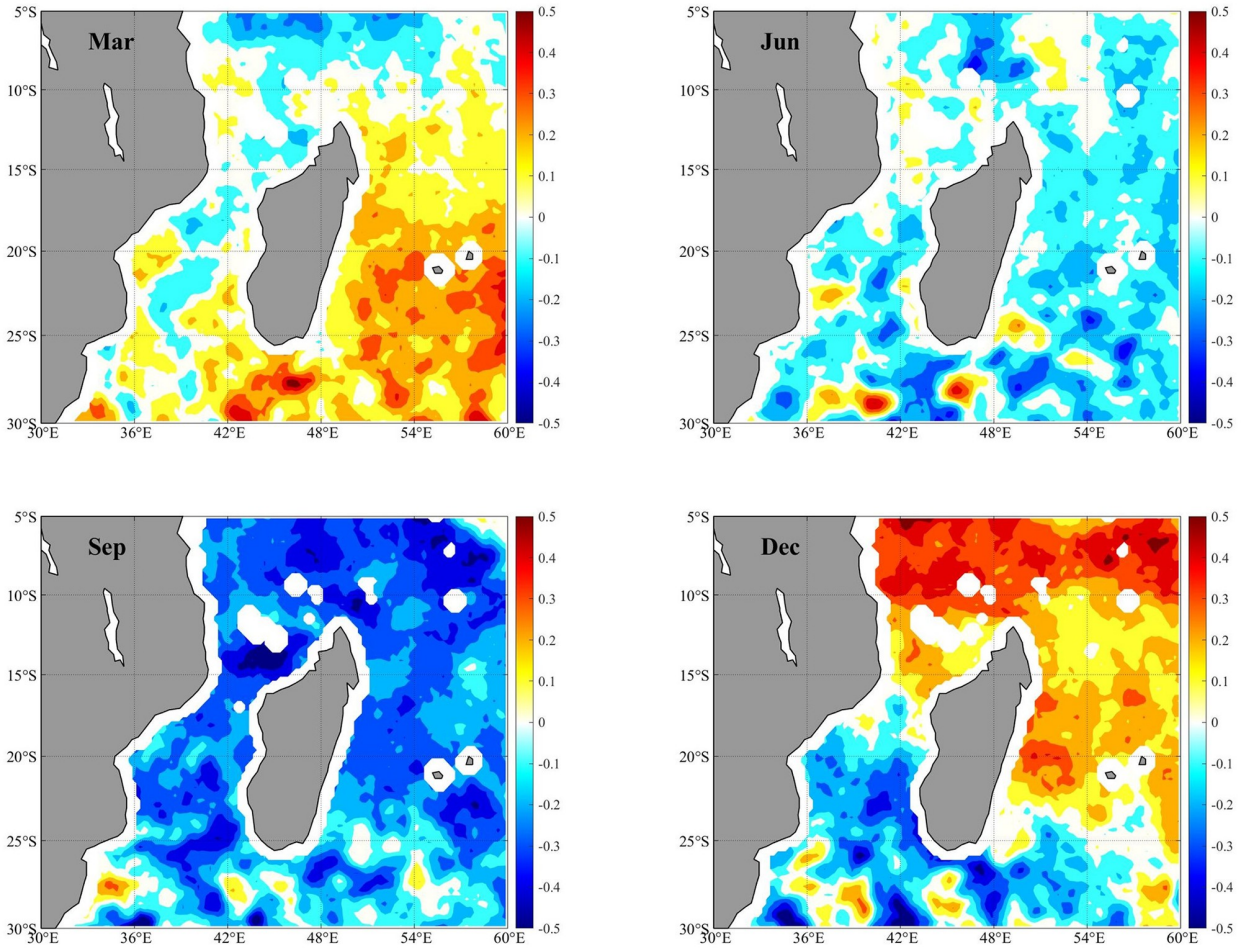
The monthly average ΔSST distribution in March, June, September and December.

Fig 9 shows the isotherm distribution of the average ΔSST for March, June, September, and December during a ten-year period. The northern Mozambique Channel is characterized by a warm pool, and we have observed a large number of eddies, whose contribution to seasonal heat transport in the channel cannot be ignored [36]. From March to June, the number of anticyclonic eddies increased, and a distinct thermal anomaly appeared locally in the Mozambique Channel (Fig 9a and 9b). In previous studies, the centers of anticyclonic eddies tended to exhibit high isotherms, characterized by significantly higher SSTs than the surrounding waters [1]. The Mozambique Channel showed a significant cooling feature in September (Fig 9c), which may have been due to the influence of the northeast ocean current and the strengthening monsoon in Madagascar. The number of anticyclonic eddies decreased and the number of cyclonic eddies increased rapidly and the SST in the strait may have been directly affected by the cooling effect of the cyclonic eddies (Fig 9c).

As the frequency of cyclonic and anticyclonic eddies changes (Fig 5), the transport of warm and cold water in the Mozambique Channel also changes. Previous studies confirmed that anticyclonic eddies can increase SST, and the uplifted cold water caused by the upwelling of cyclonic eddies can decrease SST [24,37]. Therefore, the increase in the number of anticyclonic(cyclonic) eddies could result in the abnormal rise(cooling) of SST in the Mozambique Channel.

## Conclusions

The characteristics of the eddy in the Mozambique Channel are analyzed by the algorithm of the automatic eddy detection tracker based on satellite altimeter data from 2010 to 2019. A total of 1,086 eddies are identified in the Mozambique Channel, comprising 509 cyclonic eddies and 577 anticyclonic eddies. The anticyclonic eddy has a larger amplitude and radius. In addition, due to the combined influence of monsoon patterns and ocean currents, the occurrence of anticyclonic eddies in the Mozambique Channel exhibits a higher frequency during winter compared to summer, while cyclonic eddies display an opposite trend. The seasonal patterns of anticyclonic and cyclonic eddy occurrences are inversely related. The local wind forcing has a significant effect on the formation of mesoscale eddies in the Mozambique Channel, and the northern monsoon and the southern southeast trade wind forcing promote the formation of mesoscale eddies in the Mozambique Channel in winter. The results show that the variation in mesoscale anticyclonic and cyclonic eddies in the Mozambique Channel can play an important role in the seasonal abnormal fluctuation in local SST.
